# A Transformation and Genome Editing System for Cassava Cultivar SC8

**DOI:** 10.3390/genes13091650

**Published:** 2022-09-14

**Authors:** Ya-Jie Wang, Xiao-Hua Lu, Xing-Hou Zhen, Hui Yang, Yan-Nian Che, Jing-Yi Hou, Meng-Ting Geng, Jiao Liu, Xin-Wen Hu, Rui-Mei Li, Jian-Chun Guo, Yuan Yao

**Affiliations:** 1 Key Laboratory of Biology and Genetic Resources of Tropical Crops, Ministry of Agriculture, Institute of Tropical Bioscience and Biotechnology, Chinese Academy of Tropical Agricultural Sciences, Haikou 571101, China; 2 Key Laboratory for Biology and Genetic Resources of Tropical Crops of Hainan Province, Hainan Institute for Tropical Agricultural Resources, Haikou 571101, China; 3 School of Life Sciences, Hainan University, Haikou 570228, China; 4 San Yan Research Institute, Chinese Academy of Tropical Agricultural Sciences & Hainan Yazhou Bay Seed Lab, Sanya 572025, China

**Keywords:** cassava, CRISPR/Cas9, efficient transformation, friable embryogenic calli, homozygous, SC8

## Abstract

Cassava starch is a widely used raw material for industrial production. South Chinese cassava cultivar 8 (*Manihot esculenta* Crantz cv. SC8) is one of the main locally planted cultivars. In this study, an efficient transformation system for cassava SC8 mediated with *Agrobacterium* strain LBA4404 was presented for the first time. Cassava friable embryogenic calli (FECs) were transformed through the binary vector pCAMBIA1304 harboring *GUS-* and *GFP-*fused genes driven by the *CaMV35S* promoter. The transformation efficiency was increased in the conditions of *Agrobacterium* strain cell infection density (OD_600_ = 0.65), 250 µM acetosyringone induction, and *agro*-cultivation with wet FECs for 3 days in dark. Based on the optimized transformation protocol, approximately 120–140 independent transgenic lines per mL settled cell volume (SCV) of FECs were created by gene transformation in approximately 5 months, and 45.83% homozygous mono-allelic mutations of the *MePDS* gene with a *YAO* promoter-driven CRISPR/Cas9 system were generated. This study will open a more functional avenue for the genetic improvement of cassava SC8.

## 1. Introduction

Cassava (*M. esculenta* Crantz) is an important food crop in the tropics. The tuber roots of cassava are rich in starch that is used as a staple food for 700 million people in 105 countries. Cassava provides food for humans and raw materials for industrial production, such as biofuel processing, paper products, starch processing, livestock feeds, the textile industry, and medical products [1]. Enhancing starch accumulation, increasing nutrient content, and improving disease resistance are important goals for cassava breeding. Due to cassava’s highly heterozygous genome and its low flowering and fruit setting rates [2,3], it takes longer to improve the characteristics of cassava cultivars by hybrid breeding technology.

Transgenic technology is considered a powerful tool for the genetic improvement of cassava. *Agrobacterium*-mediated gene transformation or editing in the model cultivar (*M. esculenta* cv TMS60444) has been used to modify cassava properties such as enhanced starch accumulation and modified starch functional properties [4,5,6,7], insect and disease resistances [8,9], abiotic stress tolerance [10,11], increased nutrients [12,13], and delayed post-harvest physiological deterioration (PPD) [14,15]. However, all these studies were performed on model cassava cv TMS60444, which is amenable to gene transformation. Still, it has a low tuber root yield, low nutritional quality, high viral and bacterial disease sensitivity, and no sense for farming plants [16]. Because the production of FECs is limited by cassava genotype and the factors for transformation are unclear, some local cassava varieties with excellent traits cannot be genetically transformed [17].

Establishing genetic transformation systems for the leading local planting cassava cultivars is of great significance for the genetic improvement of cassava. Genetic transformation protocols for local planting cultivars in Africa and South America have been established, such as ADI 001 (the farmer-preferred cassava germplasm in Ghana and Africa) [17], TME 204 (the elite East African farmer-preferred cassava cultivar) [18], T200 (the South African industry-preferred cultivar) [19], TME14 (the landrace commonly grown in West, Central, and East Africa cultivars) [20], Verdinha (the Northeast Brazil cultivar) [21], and other CMD-resistant varieties [22]. Cassava is also an important cultivation crop, and the consumption of cassava starch is widespread in China. Therefore, developing effective high-throughput genetic transformation procedures for popular cassava varieties in China is necessary. South Chinese cassava cultivar 8 (*M*. Crantz cv. SC8) is one of the main locally planted cassava cultivars in China [23]. It has excellent characteristics, such as a high yield (38–45 t/ha), high starch content (30–32%), strong adaptability, tolerance to fertilizer, poor soil, drought stress, and typhoon resistance [24]. It has been reported that SC8 is the best-suited genotype for industrial applications among seven Cassava South China cultivars (SC205, SC5, SC6, SC7, SC8, SC9, and SC10) [23]. Previous studies on cassava SC8 mainly focused on the induction of somatic embryos (SEs)/friable embryogenic calli (FECs) and the exploration of genetic transformation, but no positive transgenic plants were obtained despite several attempts [25,26]. We also established a method for SEs and FECs of cassava SC8 in previous studies [27,28]. Based on this, in the present study, the factors that influence transformation efficiency were optimized, such as the density of *Agrobacterium* cells, cocultivation time, FECs’ humidity, and concentration of acetosyringone (AS). An efficient *Agrobacterium*-mediated gene transformation protocol for cassava SC8 has been developed.

The CRISPR/Cas9-mediated gene editing system is an important tool for crop breeding. However, this technology has rarely been used in cassava breeding and gene function research. First, in 2017, Odipio et al. reported on a case of CRISPR/Cas9-mediated editing of the *MePDS* gene in the genome of TME 204 and cv TM60444 cassava cultivars [29]. Eight cassava genes (*nCBP-1*, *nCBP-2*, *GBSS*, *PTST1*, *SBE2, CYP79D1*, *CYP79D2,* and *MePDS*) and the promoter in the disease-susceptibility gene *MeSWEET10a* were knocked out by CRISPR/Cas9 technology; however, the homozygous mutations in these genes in the T0 generation were very low [5,29,30,31,32,33]. CRISPR/Cas9 technology was also used for homology repairing or targeting viral genes in cassava, but the efficiency needs to be improved [34,35]. The expression of Cas9 under the *Arabidopsis thaliana YAO* promoter (*pYAO*:hSpCas9 binary vector) was reported to increase the numbers of targeted and homozygous mutations in *A. thaliana* in comparison with Cas9 driven by the cauliflower mosaic virus (CaMV) *35S* promoter [36]. In this research, we used the *pYAO*:hSpCas9 binary vector to knock out the *MePDS* gene, which improved the efficiency of homozygous mutations in cassava SC8.

## 2. Materials and Methods

### 2.1. Production of Friable Embryogenic Calli (FECs) from Cassava SC8

Somatic embryos (SEs) from cassava SC8 were induced according to our previous research [27]. Cassava primary embryos were induced with axillary buds on CIM medium (Supplementary Table S1) including 15 mM CaCl_2_ and inoculated into fresh medium every 20 days. SEs were produced from primary embryos. The production of FECs was performed according to the protocol described by Nyaboga et al. with several modifications [20]. The mature secondary SEs (coral-shaped) under the microscope were divided into small pieces with a sterile syringe, transferred to Greshoff and Doy (GD) medium (containing 12 mg/L picloram, Supplementary Table S1) [37], and cultured in the dark at 28 °C. After 14–16 days, the newly formed FECs at the edges of the SEs were transferred to fresh GD medium and refreshed every 3 weeks for a maximum of 5 months.

### 2.2. Vector Constructions

*Agrobacterium* strain LBA4404, harboring the pCAMBIA1304 binary vector, was used to optimize the factors of transformation. The T-DNA region of plasmid pCAMBIA1304 contains the hygromycin selection marker gene (*hpt*) and the reporter gene β-glucuronidase (*gusA*) fused with a green fluorescent gene (*GFP*). Both the *hpt* and *gusA-GFP* genes were driven by the *CaMV35S* promoter.

The *pYAO*:hSpCas9 binary vector was used to establish the gene-editing system for cassava SC8. The *Cas9* gene was driven by the *pYAO* promoter, which has been reported to be highly expressed in the embryo sac, embryo, endosperm, and pollen of *Arabidopsis*. The gRNA scaffold was driven by the *Arabidopsis U6-26* promoter. The hygromycin selection marker gene (*hpt*) was driven by the *CaMV35S* promoter.

The phytoene desaturase (*MePDS*, Manes.05G193700) gene was used to quantify gene editing efficiency because its mutation can cause albino seedlings. The target side (GCGTACAAAGCTTCCCAGATAGG) was chosen according to the protocol described by Odipio et al., located in the 13th exon [29]. The gRNA sequence was added to the *Bsa* I restriction site linker sequence (Supplementary Table S2). The synthesized target upstream and downstream primers were diluted with ddH_2_O to a concentration of 10 μM, mixed equally at 98 °C for 3 min, cooled at room temperature, and then placed at 16 °C for 10 min. The *pYAO*:hSpCas9 binary vector was digested with *Bsa* I enzyme at 37 °C for 2 h, and the digested fragments were purified. The annealed primers and the purified vector fragments were ligated by T4 ligase at 16 °C for 6 h, and the ligation product was transformed into *Escherichia coli* DH5α. The positive colonies were identified by sequencing. The correct recombinant plasmid *pYAO*:hSpCas9-MePDS-gRNA was transformed into *Agrobacterium* strain LBA4404 by electroporation.

### 2.3. Effect of Agrobacterium Cell Densities for Transformation Efficiency

*Agrobacterium* LBA4404 harboring pCAMBIA1304 or *pYAO*:hSpCas9-MePDS-gRNA streaked on a YEP plate containing kanamycin 50 mg/L and rifampicin 50 mg/L was cultured overnight. A single colony was inoculated into 1 mL of YEP liquid medium containing the same antibiotics and cultured for 20–24 h at 28 °C at 200 rpm; then, all *Agrobacterium*-cultured solutions were added into 50 mL of fresh YEP liquid medium with the same antibiotics, and culturing continued until an OD_600_ of 0.75 was achieved. The *Agrobacterium* cells were collected and adjusted to a final OD_600_ variety (0.05, 0.25, 0.45, 0.65, 0.85) by GD liquid medium with 200 µM AS. The *Agrobacterium* cell solution could be used for infection after 60 min at room temperature. *Agrobacterium* cells were cocultured with FECs according to the following steps. Approximately 1 mL of the 3-month-old FECs was transferred to fresh GD liquid medium, fully dispersed with a 5 mL sterile pipette tip, shaken for 30 min at 50 rpm and 28 °C, and centrifuged at 1000 rpm. Then, the supernatant solution was completely removed. The FECs precipitates were suspended in a 1 mL GD liquid medium. Then, 10 mL of the above *Agrobacterium* cells was added and evenly mixed, cocultured for 25 min at 50 rpm 28 °C, and centrifuged for 10 min at 1000 rpm at room temperature. The supernatant solution was removed, and the *agro*-cocultured FEC tissues were transferred onto a nylon filter mesh and then placed on sterilized absorbent filter paper to remove excess bacteria and liquid. The nylon filter mesh with the *agro*-inoculated FECs was transferred onto GD medium with 200 µM AS to co-cultivate for 3 days under dark conditions at 22 °C.

### 2.4. Effect of Acetosyringone Concentrations for Transformation Efficiency

To investigate the effect of acetosyringone concentrations, the bacterial pellet was re-suspended in liquid GD medium with different AS concentrations (50 µM, 100 µM, 150 µM, 200 µM, 250 µM, 300 µM). FECs of 3 months (1 mL SCV) were inoculated with *Agrobacterium* cells (OD_600_ of 0.65, optimized concentration) harboring pCAMBIA1304. The *agro*-inoculated FEC tissues were transferred onto co-cultivation medium (GD medium supplemented with 50 µM, 100 µM, 150 µM, 200 µM, 250 µM, 300 µM AS) for 3 days under dark conditions at 22 °C.

### 2.5. Effect of Cocultivation Conditions for Transformation Efficiency

The effect of cocultivation days on transformation efficiency was evaluated. The *Agrobacterium* cell harboring pCAMBIA1304 was re-suspended in liquid GD medium supplemented with 250 µM AS (optimized concentration) adjusted to an OD_600_ of 0.65 (optimized concentration) for the transformation of FECs (1 mL SCV). The *agro*-inoculated FEC tissues were transferred onto GD medium supplemented with 250 µM AS for seven days (1 day, 3 days, 5 days, 7 days) under dark conditions at 22 °C.

To investigate the effect of FEC’s humidity for transformation efficiency, dry and wet conditions were evaluated. The *agro*-cocultured FEC tissues (OD_600_ of 0.65 for cell density, 250 µM AS) on nylon filter mesh were placed on sterilized absorbent filter paper to dry the FECs thoroughly. To the dried FECs was added 1 mL GD liquid medium to treat as wet FECs. The *agro*-inoculated FECs were transferred onto co-cultivation medium (supplemented with 250 µM AS) for 3 days (optimized) under dark conditions at 22 °C

### 2.6. Selection and Regeneration of Transgenic Plants

After coculturing, the FECs infected with *Agrobacterium* were washed 3 times with liquid GD medium containing 500 mg/L carbenicillin, transferred to a new nylon filter with absorbent paper underneath, placed on GD medium containing 250 mg/L carbenicillin, and then incubated for 7 days at 28 °C in the dark. After 7 days, the nylon filter was transferred to fresh GD medium supplemented with 250 mg/L carbenicillin and 8 mg/L hygromycin for 7 days at 28 °C in dark conditions. This step was repeated twice, with the hygromycin content gradually increasing from 15 to 20 mg/L. Afterward, the nylon filter with FECs was transferred onto MSN medium (Supplementary Table S1) supplemented with 250 mg/L carbenicillin and 20 mg/L hygromycin for 6–8 weeks under a 16/8 h photoperiod at 28 °C, and the medium was refreshed every 2 weeks until the green cotyledons became mature. The mature cotyledons were transferred to shoot-inducting medium (CEM, Supplementary Table S1) supplemented with 100 mg/L carbenicillin and 10 mg/L hygromycin, and the medium was refreshed every 10–15 days until mature shoots were grown.

The mature shoots were cut and transferred onto MS medium (Supplementary Table S1) supplemented with 50 mg/L carbenicillin and 10 mg/L hygromycin. Untransformed wild-type cassava seedlings were also cultured on MS medium with 10 mg/L hygromycin as a negative control. Two weeks after culturing, the transgenic shoots developed adventitious roots. In contrast, the nontransgenic shoots did not develop these roots.

### 2.7. Analysis of GUS and GFP Expression

To evaluate the efficiency of cassava transformation using the pCAMBIA1304 binary vector, GUS/GFP co-expressing transgenic tissues (FECs, embryos, cotyledons, and plants) were used for GUS staining or GFP detection. GUS expression in *agro*-infected FECs (last time on GD medium) was compared based on blue stained spotted FECs; somatic embryo samples were taken from the second time on MSN medium plates; cotyledon samples were taken from the first time on CEM medium plates; plant samples were taken from MS medium with hygromycin. The samples were immersed in GUS reaction buffer (Huayueyang Biotech Co., Ltd., Beijing, China, GT0391), placed in a vacuum for 4 h, and then incubated overnight at 37 °C. The tissues were washed with 70% ethanol for discoloration, and pictures were taken under a digital microscope (KEYENCE vhx-6000 from Japan). GFP fluorescence in the transformed tissues (embryos, cotyledons, plants) was visualized using a laser emitter (LUYOR-3415RG).

### 2.8. PCR Analysis

Cassava genomic DNA was extracted from the transformed and wild-type cassava using a plant DNA isolation kit (FOREGENE, CHENGDU). The *gusA* and *GFP* genes inserted in the pCAMBIA1304 vector were used to confirm the transgenic lines by PCR analysis using gene-specific primers (Supplementary Table S2), which amplified 722 bp fragments for *gusA* and 405 bp fragments for GFP. The Cas9 gene was used for confirmation by PCR analysis using gene-specific primers (Supplementary Table S2) in the transgenic lines of *pYAO*:hSpCas9-MePDS-gRNA, which amplified 876 bp fragments. Plasmid DNA of *pCAMBIA1304/pYAO*:hSpCas9-MePDS-gRNA and nontransformed plant DNA were used as positive and negative controls, respectively. The standard PCR volume was 50 µL, which consisted of a 200 ng DNA template, 10 mM of each primer, 10 mM dNTP mixture, 10× ExTaq buffer, and 2.5 units of ExTaq DNA polymerase. The reaction conditions for *gusA*, *GFP*, and *Cas9* were 95 °C for 3 min, 35 cycles of 98 °C for 20 s, 58 °C for 30 s, and 72 °C for 45 s, and a final 10 min extension at 72 °C. PCR products were run on 1% agarose gels with nucleic acid dyes and visualized under a UV transilluminator.

### 2.9. Sanger Sequencing and Hi-TOM Sequencing

A pair of specific primers was designed to amplify a 210 bp fragment that included the target site of the *MePDS* gene (Supplementary Table S2). Nontransformed plant DNA was used as a positive control. The PCR system and procedures were the same as those described above. PCR products were Sanger sequenced after detection with a 1.5% agarose gel. Sequences and sequencing peak maps were aligned with the wild-type reference sequence of the *MePDS* gene to characterize CRISPR/Cas9-induced mutations.

Gene editing frequency was analyzed using the Hi-TOM program for high-throughput mutations [38]. The samples were amplified using target-specific primers (Supplementary Table S2). According to the Hi-TOM protocol, the first round of PCR products was used as a template for the second round of PCR (barcoding PCR). All products of the second-round PCR were pooled in equimolar amounts in a tube and purified using the OMEGA gel extraction kit. The purified product was sent to Novogene for high-throughput sequencing, and the sequencing data were directly uploaded to the website (http://www.hi-tom.net/hi-tom/; accessed on 22 August 2019).

### 2.10. Statistical Analysis

The experimental data of treatments were from the six independent experiments, and data for all the parameters were analyzed by variance (ANOVA) using SAS 9.2. Duncan’s new multiple range test was used to detect significant differences between means.

## 3. Results

### 3.1. Effect of Agrobacterium Cell Concentration on Cassava SC8 Transformation

**Figure 1:**
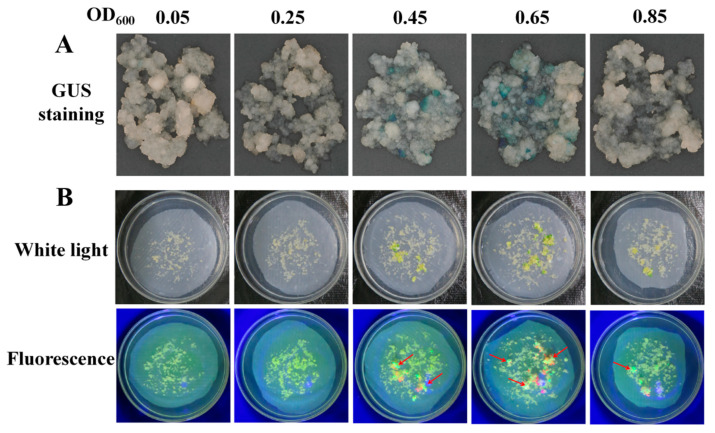
Stable expression of GUS in FECs (**A**) and GFP in cotyledons (**B**) under different *Agrobacterium* cell densities. (**A**) GUS staining of FECs. FECs were taken from the last time on GD medium plates. After GUS staining, pictures were taken under an ultradeep field microscope. (**B**) Cotyledons on MSN medium under white light and fluorescence. Red arrows point to somatic embryos with green fluorescence.

*Agrobacterium* concentration is an important factor that affects the delivery of T-DNA into plant cells. The infection efficiency for cassava SC8 FECs among 5 *Agrobacterium* cell densities (OD_600_ values of 0.05, 0.25, 0.45, 0.65, and 0.85) were analyzed. GUS staining of the 30 d-infected FECs and the number of cotyledonary-stage embryos were used to evaluate the transformation efficiency. The results showed that OD_600_ of 0.65 yielded a significantly (*p* < 0.01) higher frequency (40.00%) of GUS-positive calli and number of cotyledonary-stage embryos (79.33) than other *Agrobacterium* cell densities (Figure 1, Table 1). Furthermore, the number of GFP-positive cotyledons was investigated. The results showed that OD_600_ of 0.65 produced more GFP-positive cotyledons (Figure 1). Based on GUS expression, the numbers of matured cotyledonary-stage embryos and GFP-positive cotyledons, an *Agrobacterium* concentration OD_600_ of 0.65 was optimal for the genetic transformation of cassava SC8.

### 3.2. Effect of AS Concentration on Cassava SC8 Transformation

**Figure 2:**
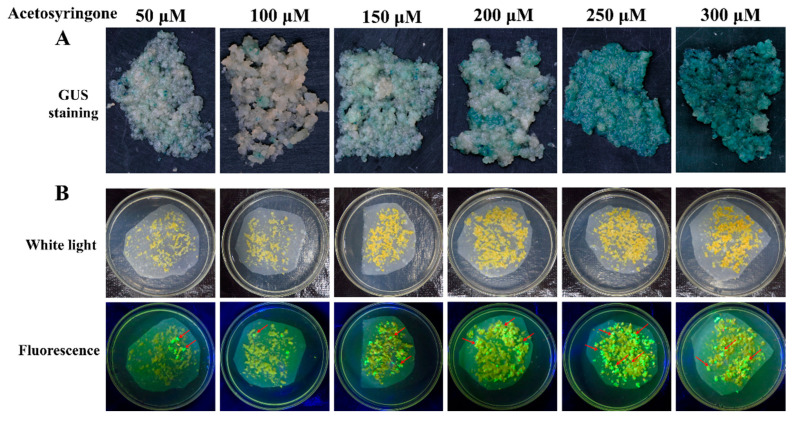
Stable expression of GUS in FECs (**A**) and GFP in cotyledons (**B**) under different concentrations of acetosyringone. (**A**) GUS staining of FECs. FECs were taken from the last time on GD medium plates. After GUS staining, pictures were taken under an ultradeep field microscope. (**B**) Cotyledons on MSN medium under white light and fluorescence. Red arrows point to somatic embryos with green fluorescence.

Acetosyringone (AS) can induce the efficient expression of the *Vir* gene in the *Agrobacterium* Ti or Ri plasmid and is an important factor for *Agrobacterium*-mediated transformation. Based on the optimal concentration of *Agrobacterium* cells, the effect of AS on the transformation of cassava SC8 was assessed at 50, 100, 150, 200, 250, and 300 µM. The results showed that supplementation with 250 µM yielded a significantly (*p* < 0.01) higher frequency (69.83%) of GUS-positive calli and the number of cotyledonary-stage embryos (114.50) than other AS concentrations (Figure 2, Table 2). At the cotyledonary stage, 250 µM AS treatment FECs yielded more GFP-positive cotyledons than other AS concentrations (Figure 2). These results showed that the optimal AS concentration for SC8 FECs transformation was 250 µM.

### 3.3. Effect of Cocultivation Conditions on Cassava SC8 Transformation

**Figure 3:**
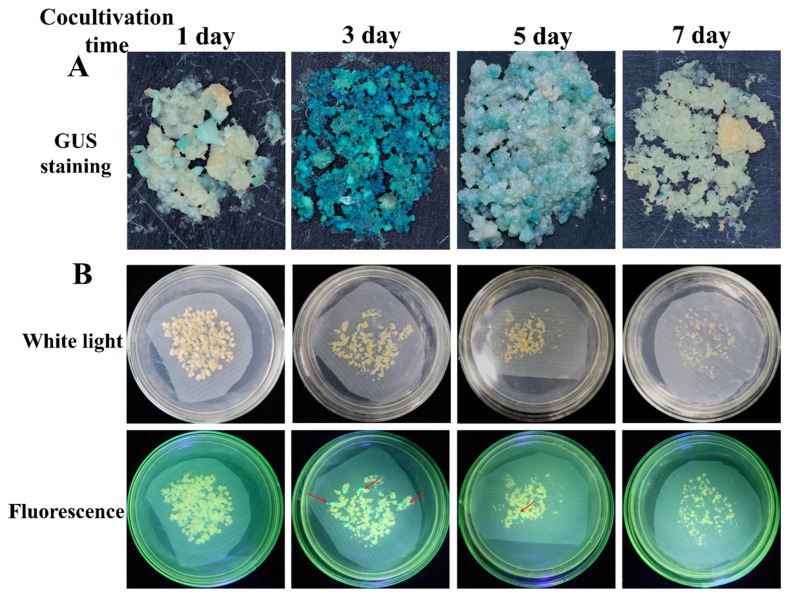
Stable expression of GUS in FECs (**A**) and GFP in cotyledons (**B**) under different cocultivation days. (**A**) GUS staining of FECs. FECs were taken from the last time on GD medium plates. After GUS staining, pictures were taken under an ultradeep field microscope. (**B**) Cotyledons on MSN medium under white light and fluorescence. Red arrows point to somatic embryos with green fluorescence.

**Figure 4:**
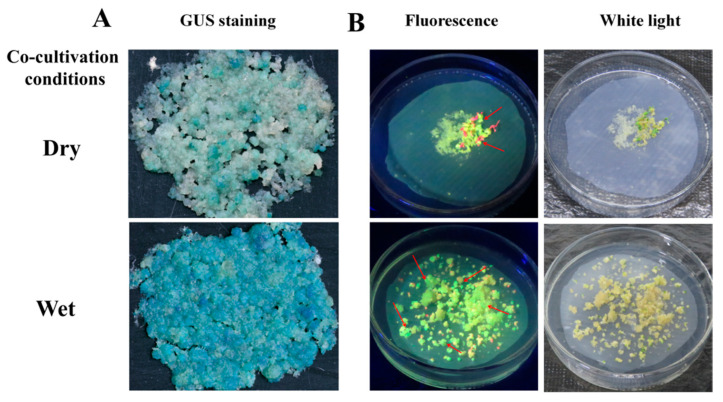
Stable expression of GUS in FECs (**A**) and GFP in cotyledons (**B**) under FECs with dry or wet treatment. (**A**) GUS staining of FECs. FECs were taken from the last time on GD medium plates. After GUS staining, pictures were taken under an ultradeep field microscope. (**B**) Cotyledons on MSN medium under white light and fluorescence. Red arrows point to somatic embryos with green fluorescence.

Cocultivation conditions, such as days of cocultivation and the FECs’ humidity, are important factors that need to be optimized in *Agrobacterium*-mediated transformation systems. Based on the optimal concentration of *Agrobacterium* cells and AS concentration, the effect of cocultivation days was co-cultivated for 1 d, 3 d, 5 d, and 7 d. The frequency of GUS-positive calli and the number of cotyledonary-stage embryos under the three-day cocultivation period were significantly (*p* < 0.01) higher than in the other periods (Figure 3, Table 3). There were the most GFP-positive cotyledons under three days’ cocultivation. The frequency of GUS-positive calli and the numbers of cotyledon-stage embryos and GFP-positive cotyledons were higher on the wet FECs than on the dry FECs (Figure 4, Table 4). Thus, a three-day cocultivation and wet FECs could increase the transformation efficiency of cassava SC8.

### 3.4. Construction of the Optimized Cassava SC8 Transformation Protocol

**Figure 5:**
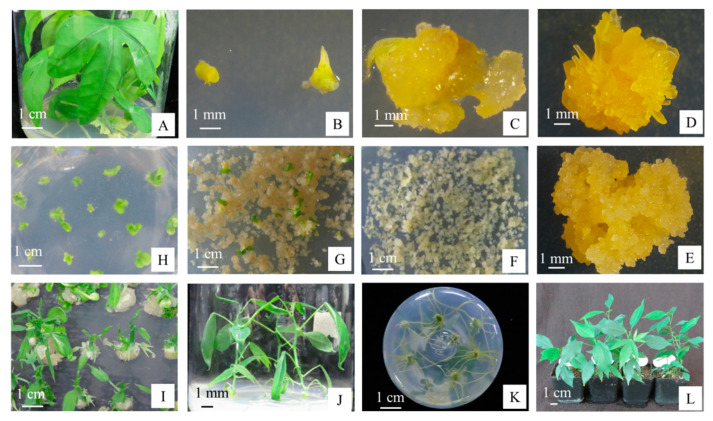
*Agrobacterium*-mediated genetic transformation of cassava SC8 FECs. (**A**) In vitro shoot culture; (**B**) axillary bud; (**C**) primary SEs on CIM medium; (**D**) SEs on CIM medium; (**E**) friable embryogenic calli on GD medium; (**F**) *Agrobacterium*-infected FECs proliferating on GD medium; (**G**) developing cotyledons on MSN medium; (**H**) cotyledons on CEM medium; (**I**) developing shoots on COM medium; (**J**) transgenic plantlets on MS medium; (**K**) rooting assay of transgenic plants on MS + 50 mg/L carbenicillin + 10 mg/L hygromycin; (**L**) transgenic plants in the soil.

**Figure 6:**
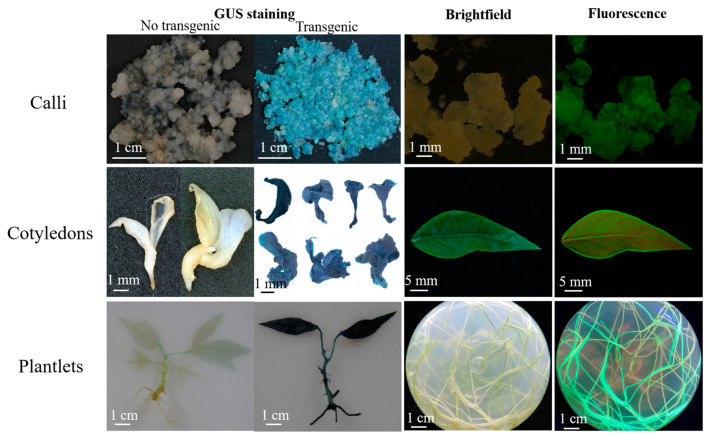
Assessments of the co-expression of *GUS* and *GFP* in transgenic tissues.

Axillary bud in vitro cassava SC8 plantlets were cultured on CIM to induce SEs (Figure 5A–D). The embryos were completely divided and placed on GD to generate sufficient amounts of FECs (Figure 5E). *Agrobacterium* strain LBA4404 harboring the binary vector pCAMBIA1304 (*GUS-* and *GFP-*fused genes driven by the *CaMV35S* promoter) was employed for FEC infection, in which *Agrobacterium* strain cell density (OD_600_ of 0.65), 250 µM AS concentration, FECs wet treatment, and a 3-day cocultivation period under dark conditions were employed (Figure 5F). The infected FECs were transformed to MSN medium with hygromycin (8, 15, 20 mg/L) to induce cotyledon initiation (Figure 5G). Mature cotyledons developed on CEM with 50 mg/L carbenicillin (Figure 5H). Mature cotyledons were enlarged, and the leaves and shoots were initially developed on COM with 50 mg/L carbenicillin (Figure 5I). The shoots were placed on MS medium with 50 mg/L carbenicillin to generate transgenic plantlets (Figure 5J). Roots were induced from the transgenic plantlets on MS with 50 mg/L carbenicillin and 10 mg/L hygromycin (Figure 5K). After molecular identification, the transgenic plants were transferred to soil (Figure 5L). Based on the above experimental protocol (Figure S1), three independent gene transformation experiments were tested for transformant efficiency by GUS staining, GFP detection (Figure 6), and PCR (Supplementary Figure S2A). The results showed that approximately 124–143 transgenic lines were generated from 1 mL SCV of *agro*-infected FECs in approximately 5 months (Table 5).

### 3.5. CRISPR/Cas9-Mediated Mutagenesis in Cassava SC8

**Figure 7:**
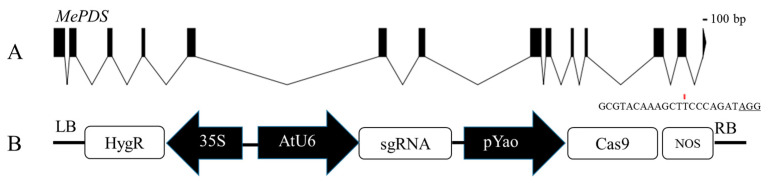
Target site of the *MePDS* gene and the T-DNA of the *pYAO*:hSpCas9-gRNA binary vector. (**A**) Structural organization of the *MePDS* gene. Exons and introns are shown as boxes and lines, respectively. (**B**) Schematic of the CRISPR/Cas9 binary vector *pYAO*:hSpCas9-MePDS-gRNA for *MePDS* gene editing through *Agrobacterium*-mediated transformation.

Using the optimized transformation system, the efficiency of CRISPR/Cas9-mediated gene editing in cassava SC8 was examined. The target side of the *MePDS* gene located in the 13th exon was chosen according to the research of Odipio et al. [29]. The *YAO* promoter-driven CRISPR/Cas9 vector *pYAO*:hSpCas9-MePDS-gRNA was constructed to mutate the *MePDS* gene, and the gRNA scaffold was driven by the *Arabidopsis U6-26* promoter (Figure 7).

**Figure 8:**
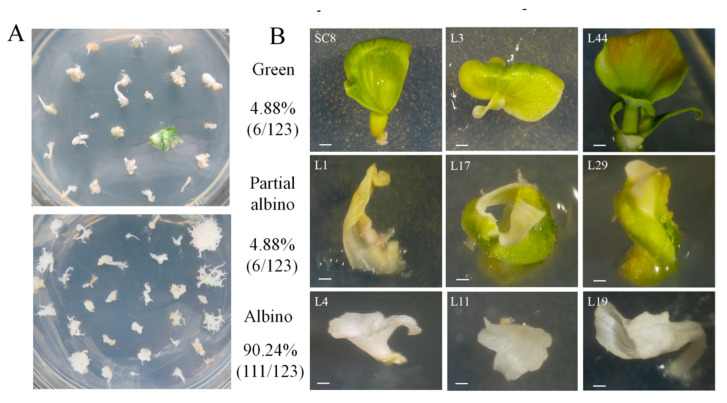
Phenotypes of the regenerated cotyledons after *MePDS* gene editing. (**A**) Regenerated cotyledons on CEM medium. (**B**) Phenotypic diversity of CRISPR/Cas9-induced *MePDS* mutations in cassava cotyledons.

The CRISPR/Cas9 vector with the target sequence was transformed into SC8 FECs using the optimized *Agrobacterium* transformation protocol. A total of 123 independent lines of the regenerated cotyledons were obtained (Figure 8A). In total, 111 cotyledons were albino (90.24%), 6 cotyledons were yellow or partially albino (4.88%), and 6 cotyledons were green (4.88%) (Figure 8B).

**Figure 9:**
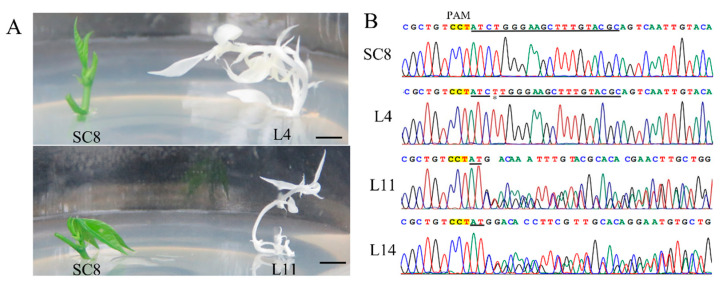
*MePDS* editing of transgenic albino SC8 cassava plants and Sanger sequences. (**A**) Albino plants and green plants of the *MePDS*-edited transgenic albino SC8 cassava. (**B**) Sanger sequences of the target sites in the *MePDS*-edited transgenic albino SC8 cassava plants.

A total of 39.02% of the regenerated cotyledon lines successfully germinated plantlets, which generated 48 independent transgenic plant lines (Figure 9A and Figure S2B). The target region (250 bp) of the *MePDS* gene from the albino plants was amplified and sequenced by the Sanger method (Figure 9B). The target site of the *MePDS* gene in the L11 and L14 lines showed a double-peak pattern, indicating that the gene editing occurred in heterozygous types. The peak pattern from Line L4 did not show a double-peak pattern, while a 1 bp insertion at the target site was adjacent to the protospacer adjacent motif (PAM).

To further analyze the mutation types of each transgenic line, high-throughput sequencing of the amplified *MePDS* gene target fragments from the 48 transgenic plant lines was performed using Hi-TOM technology [38]. Most mutations generated by editing were insertions or deletions that were usually close to the DSB site 3 bp upstream of the PAM (Supplementary Table S3). In summary, 93.75% of the *MePDS*-edited transgenic cassava SC8 plants had at least 1 mutation at the target site of the *MePDS* gene, while 6.25% had no mutation (Figure 8A). Among them, three types of homozygous mono-allelic mutations (45.83%), homozygous bi-allelic mutations (29.16%), and heterozygous mutations (18.75%) were found (Table 6). Notably, using the same target site, the homozygous mutation rate from the *CaMV35S* promoter-driven CRISPR/Cas9 vector in cassava 60,444 and TME204 was very low, and the heterozygous mutations were 66.67–77.78% (Table 6). Thus, these results demonstrated that the *pYAO*:hSpCas9 vector could efficiently create homozygous mutations in cassava. Thus, these results demonstrated that the *pYAO*:hSpCas9 vector could efficiently create homozygous mutations in cassava.

## 4. Discussion

Since the successful transformation of cassava was first reported in the 1990s, the stress resistance [11,39], nutrition [13,40], and starch properties [41,42] of cassava have been improved by transgenic technology. However, these improvements were under the cultivar cv TMS60444 background, which is unsuitable for extensive cassava cultivars. This study established an efficient genetic transformation protocol for cassava SC8, which is the main cultivar in China. *Agrobacterium* cell density is an important factor for cassava genetic transformation. Low concentrations of *Agrobacterium* might reduce the efficacy of T-DNA transformation, while high concentrations of *Agrobacterium* might affect the viability of plant cells [20]. It has been reported that optical cell densities for TME204 [18], TME14 [20], and TMS60444 [43] cassava cultivars were OD_600_ of 0.05, 0.25, and 0.50, respectively. In this study, an *Agrobacterium* cell density of OD_600_ of 0.65 was best for cassava SC8 genetic transformation. The addition of AS under cocultivation can significantly improve the transformation efficiency. A total of 6 AS concentration gradients (50–300 µM) were set up to optimize the genetic transformation system of cassava SC8. The results showed that the transgenic efficiency of cassava SC8 was highest when the AS concentration was 250 µM. No research to date has reported on the effects of the concentration of AS on genetic transformation efficiency in different cassava cultivars. In previous studies, 200 µM AS was used for the genetic transformation of farmer-preferred cassava cultivars (such as TME14, TME14, and TME204) and the model cultivar cv TMS60444. The FECs of cassava SC8 cannot be fully infected when the cocultivation period is too short (1–2 days); a 3-day cocultivation period was found to be best for cassava SC8. The cocultivation periods for other cultivars vary from 2 to 4 days [19,44]. In cassava transformation, the effect of FEC humidity under cocultivation has not been reported in previous studies. Interestingly, FECs wet conditions could increase the transgenic efficiency of cassava SC8. Based on the optimized transformation system, approximately 120–140 transgenic lines per mL SCV were regenerated for cassava SC8 in approximately 5 months. This transformation frequency is significantly higher than in previous studies that used model cultivar 60,444 and cultivars of TME14 and T200 [19,20,44].

The CRISPR/Cas9 system has been used for cassava gene editing through a genetic transformation with the *CaMV35S* promoter-driven CRISPR/Cas9 vector [29]. The frequency of homozygous mutations is important for cassava mutants. Constitutive promoters usually result in the low efficiency of homozygous mutation. It has been reported that cell division promoters (such as the *YAO* and *CDC45* promoters) for CRISPR/Cas9 gene editing could increase the yield of homozygous mutants [36]. In this research, we used the *pYAO*:hSpCas9 binary vector for the knockout of the *MePDS* gene to assess the homozygous editing rate in cassava SC8. The same target site in the *MePDS* gene was studied by Odipio et al. using the *CaMV35S* promoter-driven CRISPR/Cas9 vector [29]. The expression of Cas9 under the cell division-specific promoter *YAO* produced a mutation rate of 93.75%, which was lower than that of Cas9 driven by the constitutive promoter *CaMV35S* (100.00%). Meanwhile, a high proportion of homozygous mono-allelic mutations (45.83%) was identified from the *YAO* promoter-driven CRISPR/Cas9 vector in cassava SC8, but the proportions were only 11.11% in TMS60444 and 0.00% in TME204 when Cas9 was driven by the *CaMV35S* promoter (Table 6). It is worth noting that cassava is a highly heterozygous species, and it is difficult to obtain homozygous mutations by hybridization. This study’s high efficiency of homozygous mutations indicates that *YAO* promoter-driven CRISPR/Cas9 could be implemented as a viable approach for cassava genetic improvement.

In conclusion, the efficient genetic transformation and CRISPR/Cas9 gene editing of cassava SC8, one of the main cassava varieties in China, has been reported for the first time in this study. This method was found to efficiently generate 120–140 transgenic lines per mL SCV in approximately 5 months and up to 45.83% homozygous mono-allelic mutations by *YAO* promoter-driven CRISPR/Cas9. These results will be beneficial for the genetic improvement of cassava SC8.

## 5. Conclusions

In this study, an efficient transformation system of cassava SC8 mediated with *Agrobacterium* strain LBA4404 was presented for the first time, in which the factors of *Agrobacterium* strain cell infection (density OD_600_ = 0.65), 250 µM AS induction, and *agro*-cultivation with wet FECs for 3 days in dark conditions were found to increase transformation efficiency through the binary vector pCAMBIA1304 harboring *GUS-* and *GFP-*fused genes. Based on the optimized transformation protocol, approximately 120-140 independent transgenic lines were generated per mL SCV of FECs by gene transformation in approximately 5 months and 45.83% homozygous mono-allelic mutations of the *MePDS* gene by the *YAO* promoter-driven CRISPR/Cas9 system. This study will open a more functional avenue for the genetic improvement of cassava SC8.

## Figures and Tables

**Table 1:** Effects of *Agrobacterium* cell density on cassava SC8 transformation.

Cell Density OD_600_	Frequency of GUS Expression (%)	Average No. of Cotyledonary Stage Embryos on Selective Medium
0.05	11.83 ± 5.56 d	2.5 ± 2.07 d
0.25	12.00 ± 6.06 d	4.50 ± 2.88 d
0.45	22.67 ± 4.27 c	32.00 ± 8.02 c
0.65	40.00 ± 4.47 a	79.33 ± 10.46 a
0.85	31.33 ± 4.89 b	50.17 ± 8.50 b

Note: The values represent the means ± SD, each from six independent experiments. Different letters beside each number mean significant differences (*p* < 0.01) according to Duncan’s multiple range test (DMRT).

**Table 2:** Effects of AS concentration on cassava SC8 transformation.

Treatment	Frequency of GUS Expression (%)	Average No. of Cotyledonary Stage Embryos on Selective Medium
50 µM	11.67 ± 3.61 e	10.00 ± 5.02 d
100 µM	12.33 ± 4.63 e	16.17 ± 6.74 d
150 µM	21.67 ± 3.98 d	30.00 ± 6.81 c
200 µM	38.67 ± 4.92 c	68.33 ± 8.45 b
250 µM	69.83 ± 5.95 a	114.50 ± 7.61 a
300 µM	50.00 ± 7.21 b	78.83 ± 11.89 b

Note: The values represent the means ± SD, each from six independent experiments. Different letters beside each number mean significant differences (*p* < 0.01) according to Duncan’s multiple range test (DMRT).

**Table 3:** Effects of cocultivation period on cassava SC8 transformation.

Treatment	Frequency of GUS Expression (%)	Average No. of Cotyledonary Stage Embryos on Selective Medium
1 d	15.50 ± 5.54 c	13.17 ± 6.34 d
3 d	74.33 ± 7.99 a	122.17 ± 11.69 a
5 d	46.83 ± 5.60 b	74.00 ± 9.72 b
7 d	15.17 ± 4.31 c	44.50 ± 9.42 c

Note: The values represent the means ± SD, each from six independent experiments. Different letters beside each number mean significant differences (*p* < 0.01) according to Duncan’s multiple range test (DMRT).

**Table 4:** Effects of FECs with dry or wet treatment on cassava SC8 transformation.

Treatment	Frequency of GUS Expression (%)	Average No. of Cotyledonary Stage Embryos on Selective Medium
dry	52.33 ± 8.16 b	130.33 ± 10.88 b
wet	82.83 ± 6.08 a	196.00 ± 11.08 a

Note: The values represent the means ± SD, each from six independent experiments. Different letters beside each number mean significant differences (*p* < 0.01) according to Duncan’s multiple range test (DMRT).

**Table 5:** Validation of the optimized cassava SC8 transformation in 3 independent experiments using 1 mL SCV FECs.

Experiments	Number of Cotyledons	Number of Transgenic Plants
1	187	132
2	170	124
3	191	143

Note: Three independent experiments were performed using the optimized conditions for cassava SC8 transformation (*Agrobacterium* strain cell density OD_600_ = 0.65, 250 µM AS, and agro-cultivation with wet FECs for 3 days).

**Table 6:** Comparison of the mutation types at the same target site by *YAO* or *CaMV35S* promoter-driven CRISPR/Cas9 vector.

Variety	Promoter	Plant Lines Analyzed	Mutation Efficiency	Homozygous Mono-Allelic	Homozygous Bi-Allelic	Heterozygous
SC8 a	*YAO*	48	93.75% (45/48)	45.83% (22/48)	29.16% (14/48)	18.75% (9/14)
60444 b	*CaMV35S*	9	100.00% (9/9)	11.11% (1/9)	11.11% (1/9)	77.78% (7/9)
TME204 b	*CaMV35S*	9	100.00% (9/9)	0.00% (0/9)	33.33% (3/9)	66.67% (6/9)

Note: a, the data from this research; b, the data from Odipio et al. 2017 [29].

## Data Availability

Data are contained within the article or Supplementary Files.

## Supplementary Materials

The following supporting information can be downloaded at: https://www.mdpi.com/article/10.3390/genes13091650/s1, Figure S1: Schematic workflow of the protocol for *Agrobacterium*-mediated transformation of cassava cultivar SC8; Figure S2: Molecular analysis of transgenic plants; Table S1: The media and their compositions; Table S2: Primers used in this study; Table S3: Editing effects of *MePDS* gene by Hi-TOM analysis.
