# Supplementary material for: A Transformation and Genome Editing System for Cassava Cultivar SC8
Source: Genes (Basel). 2022 Sep 14;13(9):1650. doi: 10.3390/genes13091650 (PMC9498335; doi:10.3390/genes13091650)
Supplement: Supplementary file 1 [file genes-13-01650-s001.zip › genes-1890275-Supplementary File.pdf]

Supplementary Figures and Tables

1. Supplementary Figures

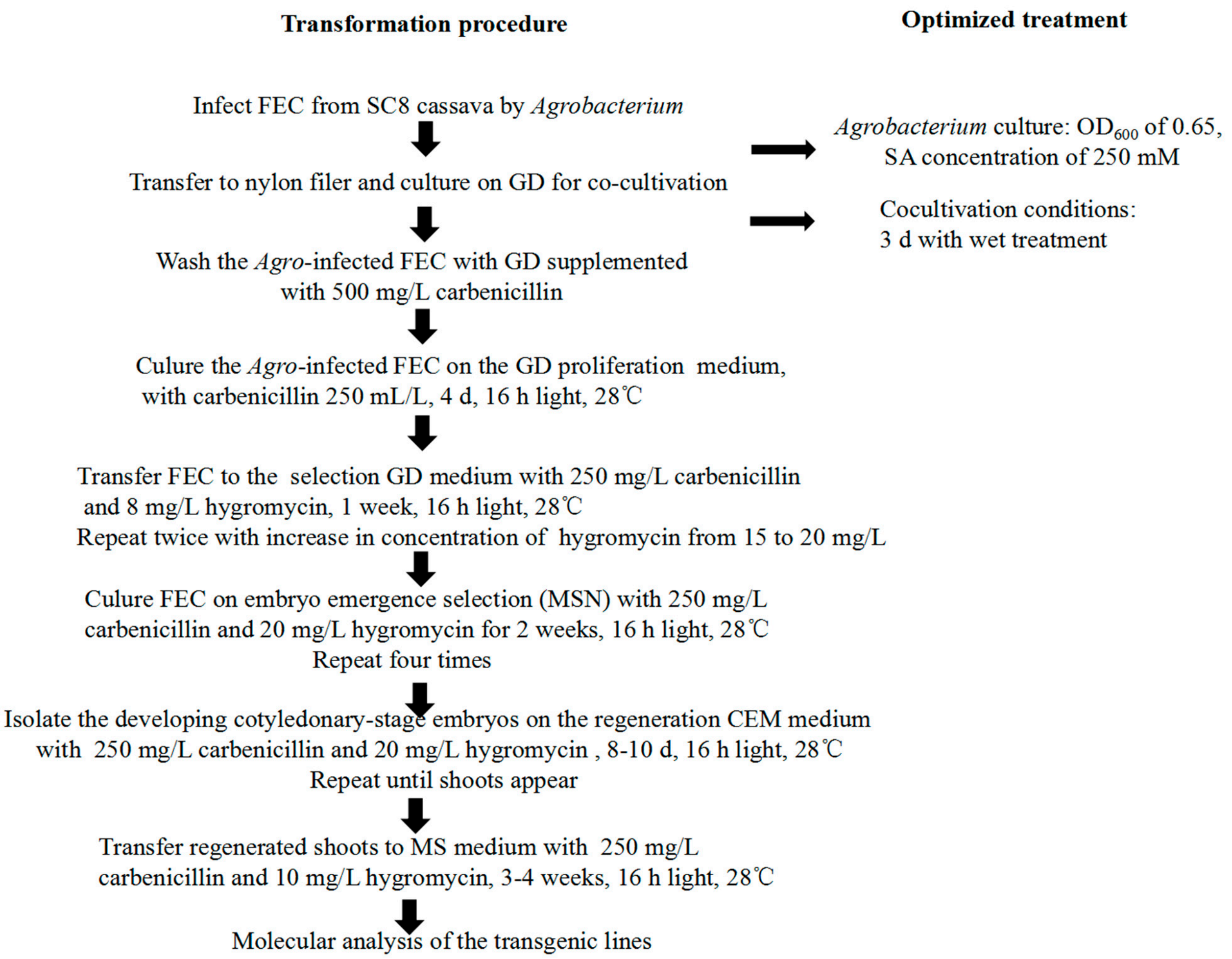

Supplementary Figure S1. Schematic workflow of the protocol for *Agrobacterium*-mediated transformation of cassava cultivar SC8. The flow chart shows the essential steps optimized and followed in the protocol.

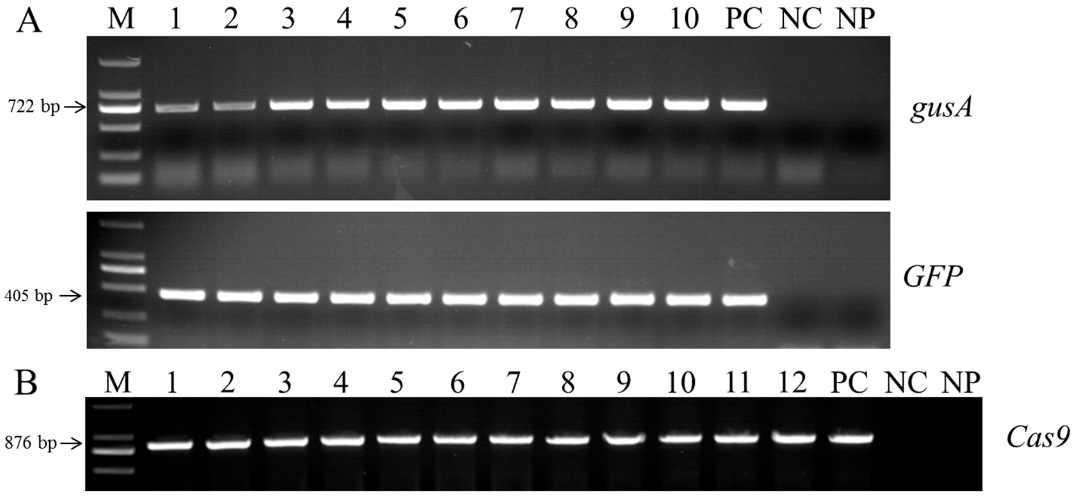

Supplementary Figure S2. Molecular analysis of transgenic plants. (A) PCR analysis of transgenic lines of cassava using *gusA* and *GFP* gene specific primers. Lanes M: 2000 bp marker (TAKARA), Lanes 1-10: different transgenic lines, PC: Positive control, NC: Negative control, NP: No template PCR. (B) PCR analysis of CRISPR/Cas9 T-DNA integration. Lanes M: 2000 bp marker (TAKARA), Lanes 1-12: different transgenic lines, PC: Positive control, NC: Negative control, NP: No template PCR.

2. Supplementary Tables

Supplementary Table S1. The media and their compositions

Medium	Compositions (1 L)
MS	4.4 g MS，30 g sucrose，0.32 mg $CuSO_4$，pH5.8，8 g Agar
GD	2.75 g GD，20 g sucrose，12 mg picloram，pH5.8，8 g Agar
CIM	4.4 g MS，20 g sucrose，0.32 mg $CuSO_4$，12 mg picloram，pH5.8，8 g Agar
CEM	4.4 g MS，30 g sucrose，0.32 mg $CuSO_4$，0.4 mg 6-BA，pH5.8，8 g Agar
COM	4.4 g MS，30 g sucrose，0.32 mg $CuSO_4$，1 mg 6-BA，pH5.8，8 g Agar
MSN	4.4 g MS，20 g sucrose，0.32 mg $CuSO_4$，1 mg NAA，pH5.8，8 g Agar

All the above culture media need to be autoclaved at 121°C for 20 min.

Supplementary Table S2. Primers used in this study.

Primer name	Primer sequence	length	Aims
MePDS-F:<br>MePDS-R:	5′-AGCTGGGGACTACACAAAGC-3′<br>5′-CCACACCCATTAGGCCTTGTAA-3′	210 bp	Amplification of the *MePDS* part
GFP-F:<br>GFP-R:	5′-CAAGGACGACGGCAACTACA-3′<br>5′-TCGTCCATGCCGAGAGTGAT-3′	405 bp	Amplification of the *GFP* part
GUS-F:<br>GUS-R:	5′-CCTCGCATTACCCTTACGCT-3′<br>5′-TTTCTTGTTACCGCCAACGC-3′	722 bp	Amplification of the *GUSA* part
CAS9-F:	5′-GCAAGCTGCTCTAGCCAATACGC-3′	876 bp	Amplification of

CAS9-R:	5′-CGGGAAACGACAATCTGATCCAAG-3′		the Cas9 part
MePDS-gRNA-F<br>MePDS-gRNA-R	5′-gattGCGTACAAAGCTTCCCAGAT-3′<br>5′-aaacATCTGGGAAGCTTTGTACGC-3′	28 bp	*MePDS* gene target
MePDS-HT-F:<br>MePDS-HT-R:	5′-ggagtgagtacggtgtgcAGCTGGGGACTACACAAAGC-3′<br>5′-gagttggatgctggatggCCACACCCATTAGGCCTTGTAA-3′	246 bp	*MePDS* gene editing test

Note: the lowercase letters represent the linker sequences.

Supplementary Table S3. Results of *MePDS* gene by Hi-TOM analysis

Lines	Sequence near the target	Reads	Ratio	Variation type	Variation sequence
**WT**	GTCCTATCTGGGAAGCTTTGTACGCAG	10974	96.26%		
**L1**	GTCCTATCTTGGGAAGCTTTGTACGCAG	766	90.22%	1I	T
	GTCCTATCTGGGAAGCTTTGTACGCAG	83	9.78%	WT	
**L2**	GTCCTATCTTGGGAAGCTTTGTACGCAG	6636	93.62%	1I	T
**L3**	GTCCTATCTGGGAAGCTTTGTACGCAG	6588	55.67%	WT	
	GTCCTATCTTGGGAAGCTTTGTACGCAG	5100	43.09%	1I	T
**L4**	GTCCTATCTTGGGAAGCTTTGTACGCAG	14197	96.76%	1I	T
**L5**	GTCCTATCTTGGGAAGCTTTGTACGCAG	13236	97.92%	1I	T
**L6**	GTCCTATCTTGGGAAGCTTTGTACGCAG	24680	84.58%	1I	T
	GTCCTATCTTGGGAAGCTTTGTACGCAG	1489	5.10%	1I,SNP	T,A->G
**L7**	GTCCTAT------------------------GTACGCAG	11295	53.29%	12D	CTGGGAAGCTTT
	GTCCTATC----GGAAGCTTTGTACGCAG	8993	42.43%	2D	TG
**L8**	GTCCTATCTTGGGAAGCTTTGTACGCAG	14194	96.76%	1I	T
**L9**	GTCCTATCTTGGGAAGCTTTGTACGCAG	12625	98.36%	1I	T
**L10**	GTCCTATCTTGGGAAGCTTTGTACGCAG	19092	94.53%	1I	T
**L11**	GTCCTAT------------------------GTACGCAG	13658	52.02%	12D	CTGGGAAGCTTT
	GTCCTATC---GGAAGCTTTGTACGCAG	11569	44.07%	2D	TG
**L12**	GTCCTATCTTGGGAAGCTTTGTACGCAG	11196	50.68%	1I	T
	GTCCTATC----------------TTTGTACGCAG	5433	24.59%	8D	TGGGAAGC
	GTCCTATC------------GCTTTGTACGCAG	5138	23.26%	6D	TGGGAA
**L13**	GTCCTATCTTGGGAAGCTTTGTACGCAG	31334	77.67%	1I	T
	GTCCTATCTTGGGAAGCTTTGTACGCAG	2770	6.87%	1I,SNP	T,A->G
**L14**	GTCCTAT------------------------GTACGCAG	185	29.60%	12D	CTGGGAAGCTTT
	GTCCTATCTTGGGAAGCTTTGTACGCAG	183	29.28%	1I	T
	GTCCTATC----GGAAGCTTTGTACGCAG	174	27.84%	2D	TG
	GTCCTATCTGGGAAGCTTTGTACGCAG	83	13.28%	WT	
**L15**	GTCCTATCTTGGGAAGCTTTGTACGCAG	6786	96.57%	1I	T
**L16**	GTCCTATCTTGGGAAGCTTTGTACGCAG	495	82.91%	1I	T
	GTCCTATCTGGGAAGCTTTGTACGCAG	102	17.09%	WT	
**L17**	GTCCTATCTTGGGAAGCTTTGTACGCAG	142	62.01%	1I	T
	GTCCTATCTGGGAAGCTTTGTACGCAG	87	37.99	WT	

L18	GTCCTAT------------------------GTACGCAG	14009	53.47%	12D	CTGGGAAGCTTT
	GTCCTATC---GGAAGCTTTGTACGCAG	11293	43.10%	2D	TG
L19	GTCCTATCTTGGGAAGCTTTGTACGCAG	12248	75.17%	1I	T
	GTCCTAT------------------------GTACGCAG	2099	12.88%	12D	CTGGGAAGCTTT
	GTCCTATC----GGAAGCTTTGTACGCAG	1665	10.22%	2D	TG
L20	GTCCTATCTTGGGAAGCTTTGTACGCAG	13550	96.58%	1I	T
L21	GTCCTATCTTGGGAAGCTTTGTACGCAG	10071	97.79%	1I	T
L22	GTCCTATCTTGGGAAGCTTTGTACGCAG	8084	85.56%	1I	T
L23	GTCCTATCTTGGGAAGCTTTGTACGCAG	20220	87.32%	1I	T
L24	GTCCTATC----------------TTTGTACGCAG	14337	47.83%	8D	TGGGAAGC
	GTCCTATC------------GCTTTGTACGCAG	13993	46.68%	6D	TGGGAA
L25	GTCCTATCTTGGGAAGCTTTGTACGCAG	26563	85.38%	1I	T
L26	GTCCTAT------------------------GTACGCAG	14442	51.77%	12D	CTGGGAAGCTTT
	GTCCTATC----GGAAGCTTTGTACGCAG	12552	45.00%	2D	TG
L27	GTCCTATCTTGGGAAGCTTTGTACGCAG	25825	92.07%	1I	T
L28	GTCCTATCTTGGGAAGCTTTGTACGCAG	23355	93.31%	1I	T
L29	GTCCTATCTTGGGAAGCTTTGTACGCAG	221	43.16%	1I	T
	GTCCTATCTGGGAAGCTTTGTACGCAG	140	27.34%	WT	
	GTCCTATC------------GCTTTGTACGCAG	77	15.04%	6D	TGGGAA
	GTCCTATC----------------TTTGTACGCAG	74	14.45%	8D	TGGGAAGC
L30	GTCCTATCTTGGGAAGCTTTGTACGCAG	27513	85.43%	1I	T
L31	GTCCTATCTTGGGAAGCTTTGTACGCAG	20529	90.14%	1I	T
L32	GTCCTATCTTGGGAAGCTTTGTACGCAG	19672	93.69%	1I	T
L33	GTCCTAT------------------------GTACGCAG	6274	51.74%	12D	CTGGGAAGCTTT
	GTCCTATC----GGAAGCTTTGTACGCAG	5246	43.27%	2D	TG
L34	GTCCTATC----------------TTTGTACGCAG	9147	50.13%	8D	TGGGAAGC
	GTCCTATC------------GCTTTGTACGCAG	8709	47.73%	6D	TGGGAA
L35	GTCCTAT------------------------GTACGCAG	12936	52.24%	12D	CTGGGAAGCTTT
	GTCCTATC---GGAAGCTTTGTACGCAG	11159	45.06%	2D	TG
L36	GTCCTAT------------------------GTACGCAG	8962	52.69%	12D	CTGGGAAGCTTT
	GTCCTATC----GGAAGCTTTGTACGCAG	7243	42.59%	2D	TG
L37	GTCCTATCTTGGGAAGCTTTGTACGCAG	28092	80.70%	1I	T
	GTCCTATCTTGGGAAGCTTTGTACGCAG	2154	6.19%	1I,SNP	T,A->G
L38	GTCCTAT------------------------GTACGCAG	11409	46.01%	12D	CTGGGAAGCTTT
	GTCCTATC---GGAAGCTTTGTACGCAG	8189	33.03%	2D	TG
	GTCCTATCTTGGGAAGCTTTGTACGCAG	5029	20.28%	1I	T
L39	GTCCTATCTGGGAAGCTTTGTACGCAG	23432	90.57%	WT	
L40	GTCCTATCTTGGGAAGCTTTGTACGCAG	21323	89.51%	1I	T
L41	GTCCTATC------------GCTTTGTACGCAG	9595	50.00%	6D	TGGGAA
	GTCCTATC----------------TTTGTACGCAG	9308	48.50%	8D	TGGGAAGC
L42	GTCCTATCTTGGGAAGCTTTGTACGCAG	24741	85.13%	1I	T
L43	GTCCTATC--GGGAAGCTTTGTACGCAG	10392	49.40%	1D	T

	GTCCTATCTTGGGAAGCTTTGTACGCAG	10150	48.25%	1I	T
**L44**	GTCCTATCTGGGAAGCTTTGTACGCAG	18162	91.12%	SNP	T->A
**L45**	GTCCTATCTTGGGAAGCTTTGTACGCAG	281	49.13%	1I	T
	GTCCTATCTGGGAAGCTTTGTACGCAG	135	23.60%	WT	
	GTCCTATC----GGAAGCTTTGTACGCAG	78	13.64%	2D	TG
	GTCCTAT-----------------------GTACGCAG	78	13.64%	12D	CTGGGAAGCTTT
**L46**	GTCCTATCTTGGGAAGCTTTGTACGCAG	144	53.33%	1I	T
	GTCCTATCTGGGAAGCTTTGTACGCAG	126	46.67%	WT	
**L47**	GTCCTATCTTGGGAAGCTTTGTACGCAG	373	53.52%	1I	T
	GTCCTATCTGGGAAGCTTTGTACGCAG	324	46.48%	WT	
**L48**	GTCCTATCTGGGAAGCTTTGTACGCAG	11013	98.55%	WT	

Note: The yellow areas are the PAM areas, the underlines represent the target sequences; The red bases represent the inserted bases, -- represent the deleted bases, Lowercase letters stand for substitution bases; I: insertion, D: deletion, SNP:substitution, WT: wild type.
